# A collision prediction framework for noncoplanar radiotherapy planning and delivery

**DOI:** 10.1002/acm2.12920

**Published:** 2020-06-19

**Authors:** Naveed Islam, Josh Kilian‐Meneghin, Steven deBoer, Matthew Podgorsak

**Affiliations:** ^1^ State University of New York at Buffalo Buffalo NY USA; ^2^ Roswell Park Cancer Institute Buffalo NY USA

**Keywords:** collision prediction, noncoplanar radiation therapy, patient safety, treatment planning

## Abstract

**Purpose:**

Noncoplanar radiotherapy can provide significant dosimetric benefits. However, clinical implementation of such techniques is not fully realized, partially due to the absence of a collision prediction tool integrated into the clinical workflow. In this work, the feasibility of developing a collision prediction system (CPS) suitable for integration into clinical practice has been investigated.

**Methods:**

The CPS is based on a geometric model of the Linear Accelerator (Linac), and patient morphology acquired at the simulator using a combination of the planning CT scan and 3‐D vision camera (Microsoft, Kinect) data. Physical dimensions of Linac components were taken to construct a geometric model. The Linac components include the treatment couch, gantry, and imaging devices. The treatment couch coordinates were determined based on a correspondence among the CT couch top, Linac couch, and the treatment isocenter location. A collision is predicted based on dot products between vectors denoting points in Linac components and patient morphology. Collision test cases were simulated with the CPS and experimentally verified using ArcCheck and Rando phantoms to simulate a patient.

**Results:**

For 111 collision test cases, the sensitivity and specificity of the CPS model were calculated to be 0.95 and 1.00, respectively. The CPS predicted collision states that left conservative margins, as designed, relative to actual collision locations. The average difference between the predicted and measured collision states was 2.3 cm for lateral couch movements. The predicted couch rotational position for a collision between the gantry and a patient analog differed from actual values on average by 3.8°. The magnitude of these differences is sufficient to account for interfractional patient positioning variations during treatment.

**Conclusion:**

The feasibility of developing a CPS using geometric models and standard vector algebra has been investigated. This study outlines a framework for potential clinical implementation of a CPS for noncoplanar radiotherapy.

## 1 INTRODUCTION

The primary objective in radiation therapy is to maximize dose to the target, while minimizing dose to surrounding healthy tissue and organs at risk. Treatment planning has evolved over the years to better achieve this objective by taking advantage of additional degrees of freedom in beam delivery. The introduction of dynamic MLC leaves allowed for not only better coverage of the target while shielding of organs at risks but also allowed modulation of beam intensity (as in IMRT).^1^ Subsequently, volumetric modulated arc therapy (VMAT) introduced an additional degree of freedom by allowing the ability to deliver radiation while the gantry is rotating with variable speed and dose rate to provide a more desirable dose distribution.^2^ The use of noncoplanar treatment beams could be considered the next step in this evolution of treatment techniques. Noncoplanar treatments are enabled by using various couch angles (in the range of 0° to ± 90°) in combination with a range of gantry angles. Although treatment fields involving couch rotations are currently used in a limited capacity for cranial treatment sites, the full potential of noncoplanar radiation therapy has not been realized. A properly designed noncoplanar approach would allow an increase in the number of independent beam paths to the target, and consequently could help spread the unwanted dose spillage to a larger volume of lower dose while potentially escalating dose to the target. The feasibility of achieving a better dose distribution with optimized noncoplanar beams have been demonstrated in several studies to reduce dose to surrounding critical structures while depositing the desired dose to the target volume^3^, ^4^, ^5^, ^6^, ^7^, ^8^, ^9^, ^10^, ^11^, ^12^, ^13^. However, for clinicians to confidently incorporate such advanced techniques into their clinical practice, a reliable collision prediction system during the treatment planning process is essential.

Since the very early days of C‐arm Linacs, patient safety as well as equipment safety has been a concern due to potential collisions of the rotating gantry with the treatment couch or patient. Traditionally, treatment planners and therapists would conduct a dry run prior to plan delivery. With increasing complexity of treatment plans the need for a collision avoidance system has become more relevant recently, especially with increased prevalence of stereotactic body radiotherapy (SBRT) and stereotactic radiosurgery (SRS) treatments. Stereotactic treatments often involve the use of couch kicks, specialized immobilization devices, and in some cases stereotactic cones which can result in tight clearances.

Collision detection and avoidance in radiation therapy has been explored using different approaches over the last several decades.^14^, ^15^, ^16^, ^17^, ^18^, ^19^, ^20^, ^21^, ^22^, ^23^, ^24^, ^25^, ^26^, ^27^ Some of the earlier works on collision detection involved using geometric analytical models of the Linac and treatment couch but did not provide a realistic method for incorporating patient‐specific models. ^17^, ^19^, ^20^, ^21^, ^23^, ^25^, ^26^ Some other approaches focused primarily on graphical treatment simulation user interfaces involving generic patient models or partial body contours from planning CT scans.^18^, ^22^, ^24^, ^27^ More recent works on collision detection, however, have involved more complex approaches. Yu et al. have used a highly detailed computer‐assisted design model of the linear accelerator and treatment couch while using a hand‐held 3D scanner to capture patient surface anatomy.^14^ Both Padilla and Cardan have used multiple Microsoft Kinect cameras to acquire patient‐specific models.^15^, ^16^

Previous works on collision prediction either did not or only partially addressed all practical aspects of integrating noncoplanar radiotherapy into clinical workflow. None of the previous literature has explicitly mentioned the need for a couch coordinate prediction methodology in order for the collision prediction tool to effectively provide the treatment planner the collision‐free treatment delivery space. In addition, it is not clear how some of these previously developed collision prediction tools account for interfractional changes in patient positioning on the treatment couch. Furthermore, one of the major aspects of noncoplanar delivery and workflow that has not been considered in previous works is the need for imaging to verify patient positioning after a couch rotation has been applied.

The primary goal of this work is to develop a general framework for collision prediction that addresses some of the key challenges with integrating noncoplanar radiotherapy into clinical workflow. In this work we investigate the feasibility of a specific implementation of the developed framework through the use of a single Microsoft Kinect Camera, in combination with CT scan images to model the patient set‐up geometry, and geometric information of various Linac components to develop a CPS. Methods have been explored to determine the Linac couch coordinates based on the selection of a treatment isocenter. In the context of noncoplanar treatment delivery workflow, to address the need for imaging capabilities to verify patient positioning after couch has been rotated, the imaging components for both MV and kV systems have been modeled.

## 2 Materials and methods

**Fig. 1:**
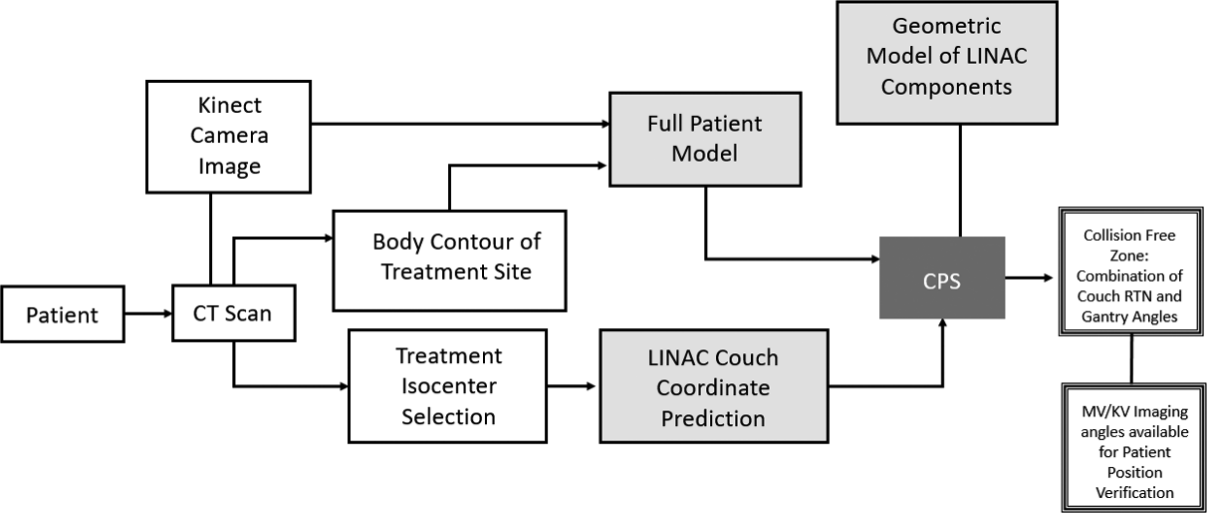
Flow chart of CPS Framework. The full patient model uses a combination of the body contour from the patient’s planning CT scan and a Kinect Camera Image acquired sequentially. Using a standardized patient set up protocol and radio‐opaque markers embedded in the CT couch top, Linac couch coordinates required to align the patient with the treatment Isocenter are calculated. Physical measurements of the Linac gantry, treatment couch, kV source head, and kV and MV imaging panels are used to construct geometric models for the CPS. The output of the CPS is provided in terms of collision free gantry angles for different couch rotational positions.

The CPS framework has been developed with the intent of making it compatible with the expected clinical workflow. A practical CPS must include the following characteristics: (a) model treatment delivery components accurately, (b) acquire patient set‐up geometry during simulation, (c) predict the treatment couch coordinates based on the correspondence between the CT couch, Linac couch, and the beam isocenter selected during the plan development, (d) provide collision information for verification imaging following each couch position adjustment, and (e) account for day‐to‐day variation in patient set up on the treatment couch. An overview of the various elements of the CPS process flow is illustrated in the diagram in Fig. 1. The details of the various components and steps in the implementation of the system have been described in the following sections.

### 2.A CPS geometric model of Linac and couch

**Fig. 2:**
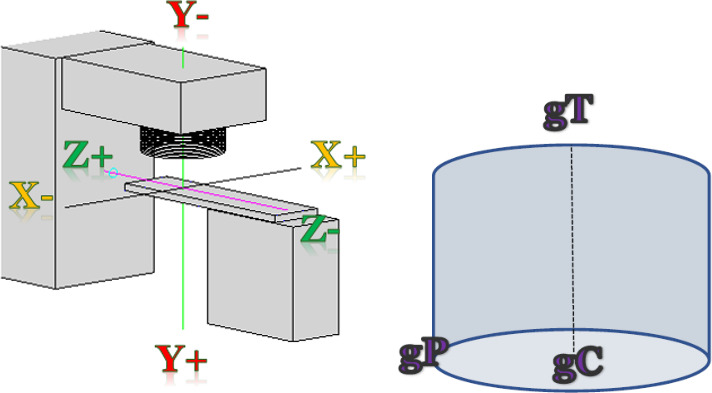
(Left) Geometric model of the Linac with IEC coordinate covention (Right) Example of a cylinder used to model part of gantry head: gP, gC and gT are points used to represent the cylinder.

**Fig. 3:**
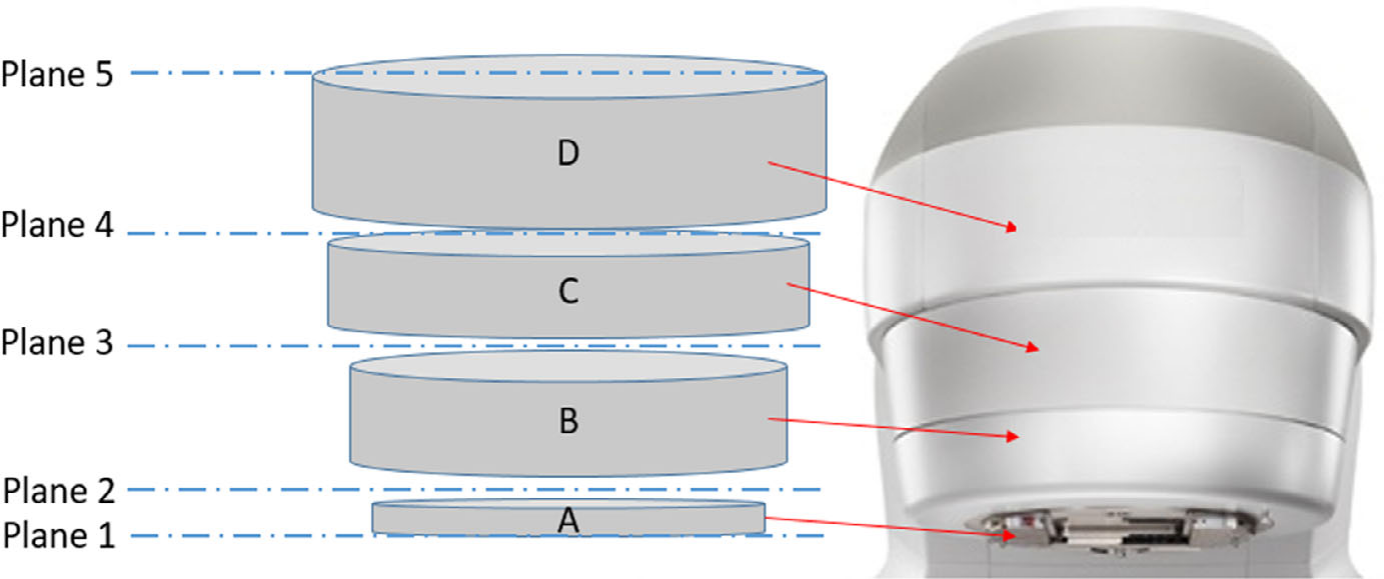
Schematic diagram of Gantry Model for CPS.

The CPS uses the coordinate system convention of the International Electrotechnique Commission (IEC), which is also used in most modern treatment planning systems. The collision model developed in this work is based on fundamental principles in geometry. Separate components of the treatment couch and linear accelerator were modeled using geometric shapes in a three‐dimensional space with the origin corresponding to the Linac isocenter. The components included the gantry and collimator head, kV source head, MV and kV Imaging panels, and the treatment couch (as shown in Fig. 2, for simplicity imaging devices are not shown). The physical dimensions of the treatment couch, gantry, MV imager, kV imager, and kV source were measured in the treatment room for a Varian True Beam STX. The treatment couch was modeled using the eight corner points of a trapezoidal prism that corresponds to the shape of the couch top. The MV‐imager, kV‐source, and kV‐Imager structures were each modeled using eight corner points to define a rectangular prism that encapsulates the corresponding structure. The gantry head has been modeled using four cylinders with a diameter corresponding to the appropriate part of the gantry head (Fig. 3). The lowest point of these set of cylinders corresponds to the lowest point on the collimator face. Each cylinder has been represented using three points: two points on the central axis and one point on the circumference (Fig. 2).

The range of allowable motions for each of the components has been incorporated into the model. To rotate the gantry by angle θ, the following rotation matrix was applied to all points used to model the gantry:

(1) $$\left[\begin{array}{ccc} \cos\left(\theta \right) & -\sin\left(\theta \right) & 0\\ \sin\left(\theta \right) & \cos\left(\theta \right) & 0\\ 0 & 0 & 1 \end{array}\right]\left[\begin{array}{c} g_{1}\\ g_{2}\\ g_{3} \end{array}\right]=\left[\begin{array}{c} g_{1}^{\prime }\\ g_{2}^{\prime }\\ g_{3}^{\prime } \end{array}\right].$$

Similarly, the following rotation matrix has been applied to all corner points used to model the couch:

(2) $$\left[\begin{array}{ccc} \cos\left(\theta \right) & 0 & -\sin\left(\theta \right)\\ 0 & 1 & 0\\ \sin\left(\theta \right) & 0 & \cos\left(\theta \right) \end{array}\right]\left[\begin{array}{c} c_{1}\\ c_{2}\\ c_{3} \end{array}\right]=\left[\begin{array}{c} c_{1}^{\prime }\\ c_{2}^{\prime }\\ c_{3}^{\prime } \end{array}\right].$$

Note that for both equations above, the rows, columns, and coordinates correspond to the CPS coordinate system displayed in Fig. 2. The longitudinal, lateral, and vertical positions of the couch were shifted by applying the following equation to all points, p, used to model the couch:

(3) $$p^{\prime }=p+mD$$

where m represents the magnitude of the shift required and D is a unit direction vector derived from a pair of points used to model the couch that correspond to the direction of the couch motion. The CPS model has been implemented using MATLAB version 2018 (Mathworks, Natick, MA, USA).

### 2.B CPS patient model: Kinect camera data calibration and coordinate transformation

**Fig. 4:**
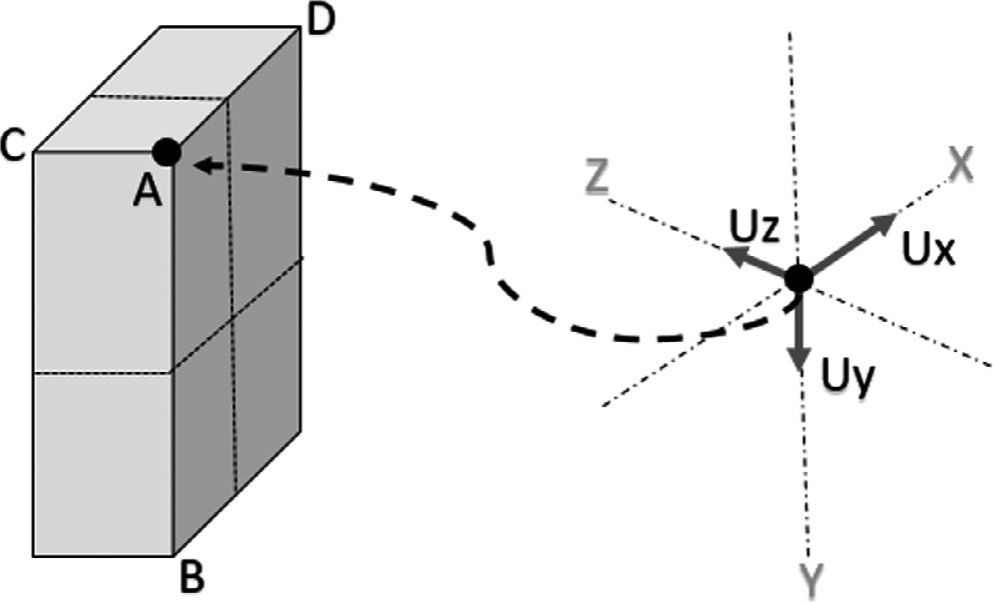
Illustration of the coordinate transformation and scaling applied to raw Kinect Camera data prior to importing into CPS.

The patient‐specific model is constructed using a combination of body contours extracted from the planning CT and surface images acquired using a Kinect^TM^ Camera V2 (Microsoft, CA, USA). The planning CT body contour is used in addition to the Kinect Camera because it is more accurate and subsequently serves as a baseline for the full patient model. The Kinect Camera has an infrared source and sensor that gives the device depth sensing capability based on time‐of‐flight of infrared signals. The depth sensor on the Kinect camera has a 512 × 424 pixel resolution and a field of view of 70.6° × 60°. At a distance of 2 m from the camera, the camera is expected to have a spatial resolution of 3 mm.^28^ Using the Kinect fusion software, point clouds of the field of view have been extracted. The density of the acquired point cloud was about 300,000 points per frame.^29^ The coordinate system of the raw Kinect data was transformed and scaled so that it can be imported into the CPS model and coordinate system. A calibration procedure has been developed to help determine the appropriate coordinate transformations and scaling factors. The Kinect camera was rigidly mounted in the CT simulation room approximately less than 2 m from the laser centers and not disturbed over the duration of any Kinect scans that were acquired. The calibration procedure was developed using a plastic block with known dimensions. The plastic block was aligned with the lasers so that the set‐up point is at the center of the block. The following steps were used to determine the appropriate transformation required:

1. In the raw Kinect Camera scan data, the 3‐D coordinates of four distinct corners of the block were identified as references points A, B, C, and D (as shown in Fig. 4).
2. All points in the raw Kinect dataset were transformed, using vector subtraction so that the new origin is located at the reference point A.
3. The angular offset between the unit vector Ux and AD was determined and the corresponding rotation matrix was subsequently applied to all raw Kinect data so that Ux and AD were aligned.
4. The previous step was then repeated to align vectors AC and AB with the corresponding unit vectors Uz and Uy.
5. Since the physical distances between the reference points are known, the appropriate scaling factor was determined and applied for each corresponding dimension of the raw Kinect data.
6. Finally, the origin was then translated from reference point A to the center of block using vector subtraction.

The approach described above resulted in bringing all the points in the raw Kinect camera scan in the same coordinate system and scale as the CPS model. However, there were some minor residual errors in orientation. To solve this problem, an additional iterative closest point (ICP) algorithm was used to help register the Kinect point cloud with the CT scan body contour point cloud. All subsequent images of phantoms captured by the Kinect camera were registered by the same set of transformations as described here.

### 2.C Treatment couch coordinate prediction

The couch coordinates are predicted using a couch coordinate prediction protocol that has been developed and implemented clinically. This approach utilizes identical couch indexing to establish a correspondence between the CT and Linac couches. Special radio‐opaque markers have been embedded under the CT couch top in order to mark reference positions on the CT couch. The Linac couch coordinates corresponding to these markers on the CT couch were determined ahead of time in order to establish a mapping between the two couches. Once the dosimetrist selects the treatment isocenter, the corresponding Linac couch coordinates can be calculated based on the relative position of the radio‐opaque markers on the CT couch. This approach assumes that the patient will be set up on the CT and Linac couches in identical locations (both in lateral and longitude directions) on the couch top, with reference to the couch indices.

**Fig. 5:**
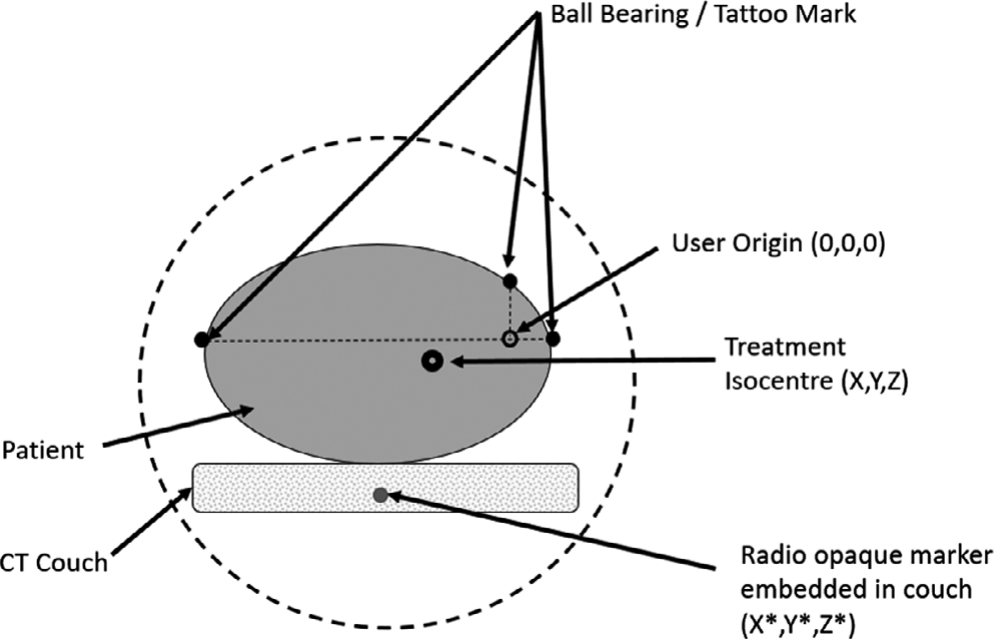
Schematic diagram of Linac couch coordinate prediction method that uses a combination of radio opaque markers in the CT couch and the selection of treatment isocenter.

The following example illustrates the principle used to predict couch coordinates. Suppose the CT scan of a patient has been acquired. Subsequently, the treatment planner selects the treatment isocenter. In order to predict the final couch coordinates on the Linac, the coordinates of one of the radio‐opaque reference markers underneath the CT couch need to be identified in the CT image sets in the treatment planning system. Suppose (X, Y, Z) denotes the location of the treatment isocenter and (X*, Y*, Z*) denotes the location of the radio‐opaque reference marker (Fig. 5). Since the reference marker has been laterally centered in the treatment couch, the couch lateral value can be predicted using the following formula:

(4) $$CouchLAT=\left\{\begin{array}{cc} 1000-\left(X-X^{*}\right) & if\;X>X^{*}\\ -\left(X-X^{*}\right) & if\;X<X^{*} \end{array}\right\}.$$

Since the vertical distance from the reference marker to the top of the CT couch top has been measured and known (e.g., 4.1 cm), the couch vertical value can be predicted using the following formula:

(5) $$CouchVRT=Y^{*}-Y-4.1.$$

Since it has been predetermined that the radio‐opaque marker corresponds to Linac couch longitude value of 140, the couch longitude value can be calculated:

(6) $$CouchLNG=140-\left(Z-Z^{*}\right).$$

Note that the method for couch LNG coordinate prediction assumes that the existing clinical workflow uses a couch indexing method to develop a direct correspondence between the patient set up in the CT sim couch and the Linac couch. Furthermore, the approach described above can be refined to account for various other factors such as couch sag and differences in placement of patient immobilization accessories from CT couch to Linac couch.

### 2.D Collision detection algorithm

**Fig. 6:**
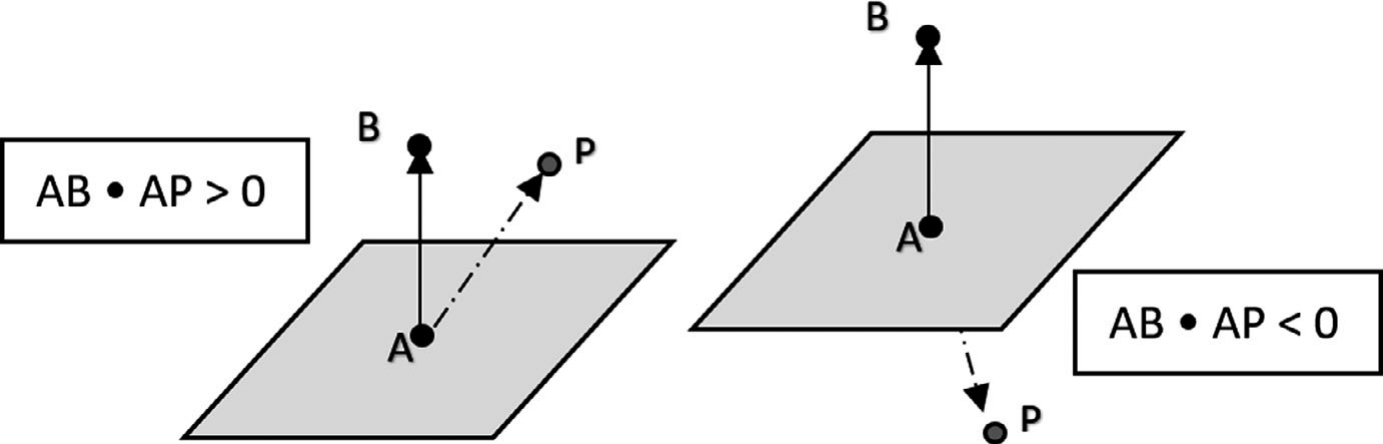
Illustration of inner (dot) product principle used in algorithm to determine collision conditions.

The CPS detects potential collision scenarios by iteratively looping through a set of points in the model and checking whether or not a given test point is inside a particular test volume in the model (e.g., cylinder used to model the collimator and gantry head). The following principle has been used in various phases of the collision detection algorithm employed by the CPS. Consider the simple problem of determining which side of a plane a particular test point, P, is located. Suppose the plane is specified with a normal vector AB (Fig. 6). Consider the following inner (dot) product: AB • AP. Since the dot product involves the cosine of the angle between the two vectors, it follows that if the test point P is on the same side of the plane as vector AB, the dot product will be positive. Conversely, if the test point is on the other side of the plane the dot product will be negative.

This application of dot product has been utilized in several stages of the collision detection algorithm used in the CPS. To determine whether or not a given test point is within a cylinder used to model part of the gantry head, the dot product approach is used to determine whether a given test point is in between the planes corresponding to the top face and bottom face of the cylinder. Subsequently if the point is found to be between those two planes, the perpendicular distance to the central axis of the cylinder is calculated. If the distance to the central axis is less than the radius of the cylinder then a collision has been detected.

In the full gantry head model consisting of four cylinders, any given test point is first tested to be in between plane 1 and plane 5 using the dot product approach (See Fig. 3). Subsequently additional dot products are taken to determine which pair of planes the test point lies in between. Depending on which region the test point is in, the radial distance to the central axis of the cylinder in question is calculated and compared with the appropriate radius.

The dot product approach is also used to detect collisions with the MV imager, kV imager, and kV source by iterating through test points and checking if any points are found to be inside a given rectangular prism modeling the corresponding device. In this case, six different dot products are evaluated to check if the given test point is inside the rectangular prism. For each face of the prism, a dot product with a normal vector, for the corresponding plane, is taken to check if the point is located on the side of the plane that contains the interior of the prism.

Furthermore, the dot product approach is also used to shorten the number of test points to iterate through when checking for collisions. The planes corresponding the inferior and superior edges of the gantry head are used to filter out points outside of the collision range of the gantry.

### 2.E Evaluation of CPS model

Each component of the CPS has been evaluated independently. End‐to‐end testing has also been conducted using Rando Phantom and ArcCheck phantom. The predictive capability of the CPS has been evaluated using the Receiver Operator Characteristic (ROC) formalism, as shown in Table 1.

**Table 1:** Receiver Operator Characteristic (ROC) formalism applied to evaluation of CPS performance.

	CPS: virtual collision	Observed physical collision
True Positive (TP)	Yes	Yes
True Negative (TN)	No	No
False Positive (FP)	Yes	No
False Negative (FN)	No	Yes

## 3 RESULTS

### 3.A Evaluation of CPS geometric models

The geometric model of various systematic components has been evaluated by comparing in room collision test cases with CPS predicted values. The experiments to evaluate accuracy of the geometric models have been preformed systematically by dividing the collision test cases into the following categories: (a) Gantry – Couch Collisions, (b) Gantry – Patient collisions, (c) MV imager – Couch/Patient Collisions, and (d) kV Source/kV imager – Couch/Patient Collisions.

#### 3.A.1 Gantry – couch collisions

**Fig. 7:**
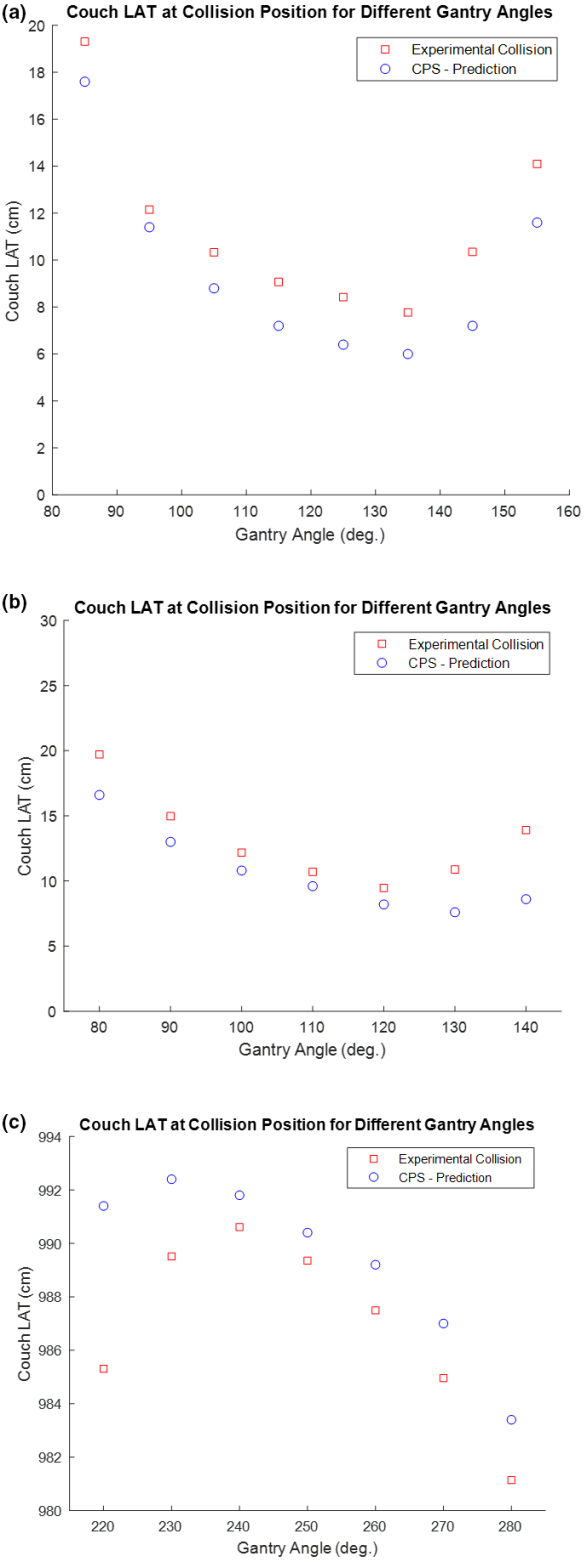
(a) Gantry‐Couch Collision Test: Couch LAT at collision position for different gantry angles. Fixed couch parameters: LNG = 85, VRT = 21.6, RTN = 0. If needed, see Fig. 8 for reference diagram of common Linac motion parameter conventions. (b) Gantry‐Couch Collision Test: Couch LAT at collision position for different gantry angles. Fixed couch parameters: LNG = 95, VRT = 16.6, RTN = 0. If needed, see Fig. 8 for reference diagram of common Linac motion parameter conventions. (c) Gantry‐Couch Collision Test: Couch LAT at collision position for different gantry angles. Fixed couch parameters: LNG = 95, VRT = 16.6, RTN = 0. If needed, see Fig. 8 for reference diagram of common Linac motion parameter.

**Fig. 8:**
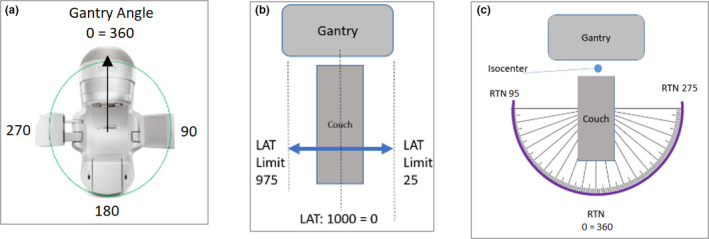
Reference diagram displaying some of the common Linac motion capabilities and motion parameter conventions: (a) Gantry motion (b) Couch lateral shift (c) Couch rotation.

**Fig. 9:**
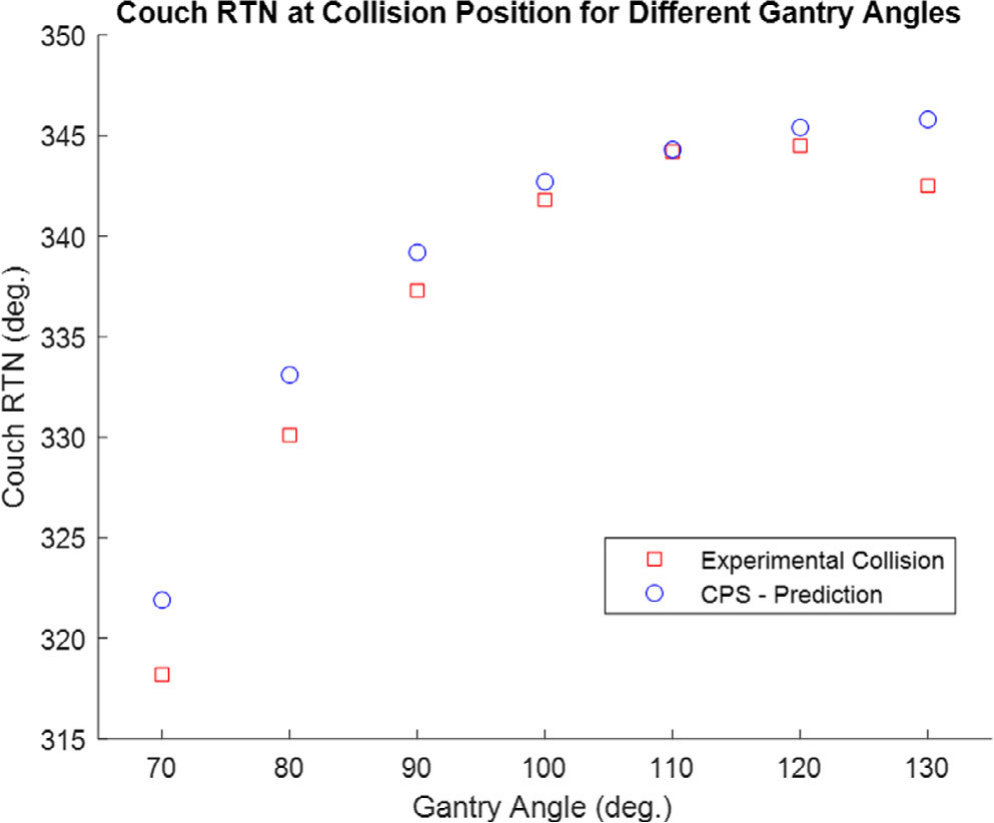
Gantry‐Couch Collision Test: Couch rotational value at collision position for various gantry angles. Fixed couch parameters: LAT = 0, LNG = 95, VRT = 16.6. If needed, see Fig. 8 for reference diagram of common Linac motion parameter conventions.

To test collisions between the gantry and couch models, the following experiments were conducted. For static gantry angles ranging from 80° to 160°, the couch was moved laterally until there was a collision. The couch lateral value in the collision position was then recorded for both the virtual model as well as the in room experiments. This experiment was done with two different preselected couch longitude, vertical and rotation parameters. The results for these sets of experiments have been displayed graphically in Figs. 7(a)–7(c) (see Fig. 8 for Linac motion parameter conventions). In both sets of experiments, the couch lateral value of the CPS predicted was at a value prior to the actual in room collision couch lateral value, indicating that CPS has a built in conservative margin. The average error in the CPS prediction was 2.37 cm. A similar experiment was repeated for static gantry positions but the couch was rotated until there was a collision (Fig. 9). Similar to previous results, the predicted couch rotational position at collision was in the conservative direction. The average error in the couch rotational positions was 2°.

#### 3.A.2 Gantry – phantom collisions

**Fig. 10:**
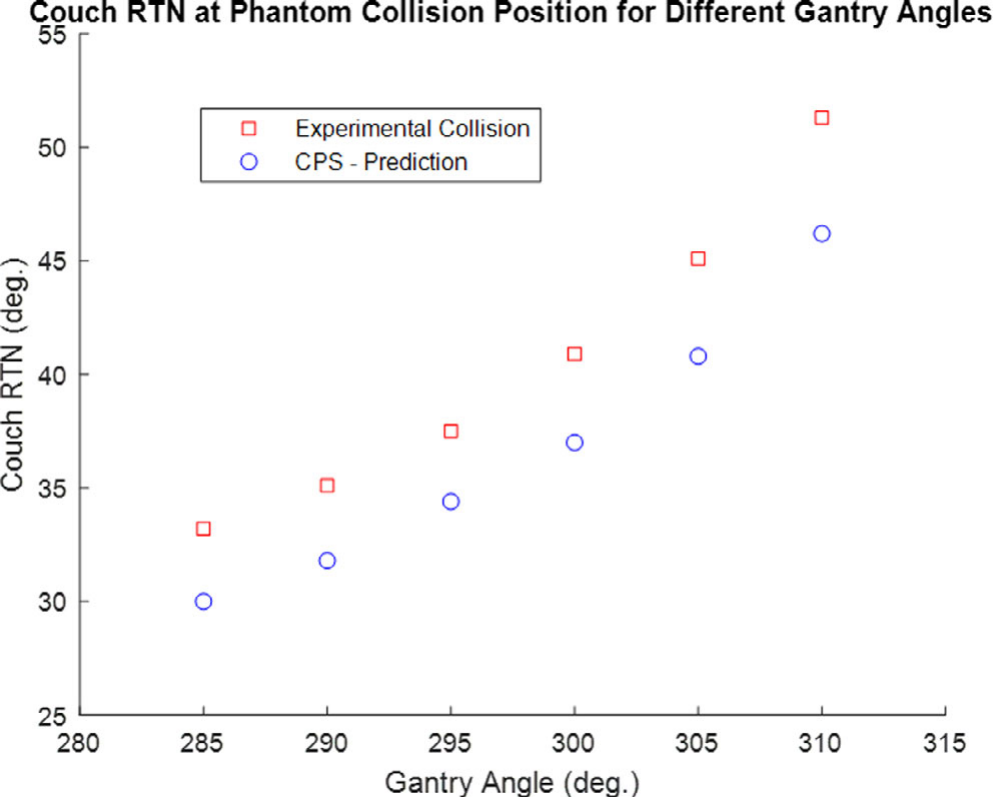
Gantry‐Couch Collision Test: Couch rotational value at collision (with Arch Check) position for various gantry angles. Fixed couch parameters: LAT = 995, LNG = 70, VRT = 21.54. If needed, see Fig. 8 for reference diagram of common Linac motion parameter conventions.

A CT scan and corresponding Kinect Camera scan were acquired for the ArcCheck phantom in order to model the ArcCheck in place of the patient in the CPS. The ArcCheck phantom was then set up in the treatment room and several collision scenarios were simulated. The couch LAT, LNG, and VRT positions were adjusted so that when the couch rotates the point of collision with the gantry occurs with the ArcCheck phantom. Similar to previous experiments, the gantry was rotated to specific angles and kept fixed. At each gantry angle the couch was rotated until there was a collision with the ArcCheck Phantom (See Fig. 10). The average error in the couch rotation value at the collision state was 3.8° with a standard deviation of 0.8°.

#### 3.A.3 MV‐imager collisions

**Fig. 11:**
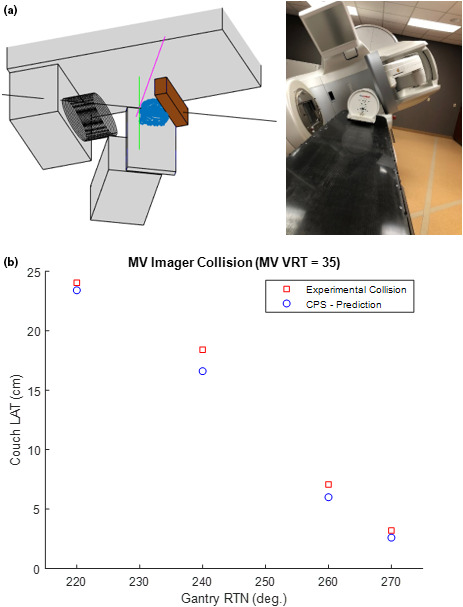
MV Imager Collision Test: (a) (Left) CPS visualization of predicted collision state with the MV imaging panel (Right) Photograph of in‐room experimental collision test case with MV imager (b) The couch LAT value at which a collision occurred with the MV imaging panel. The first two data points at gantry angles of 220 and 230 correspond to collision with ArcCheck phantom, whereas subsequent collisions occurred with the couch top. Constant couch parameters: LNG = 95, VRT = 16.54, RTN = 0. If needed, see Fig. 8 for reference diagram of common Linac motion parameter conventions.

To test the MV‐imager model, a similar type of experiment was conducted using the ArcCheck phantom positioned on the couch top. The MV‐imager panel was extended out at an arbitrary vertical position of 35 cm. The gantry was then rotated and kept fixed for angles ranging from 220 to 270, by 20° increments. For each gantry angle examined, the couch was shifted laterally until a near collision state is achieved with either the couch top or ArcCheck phantom [Fig. 11(a)] The couch lateral value at which the collision occurs was recorded and compared with the CPS’s virtually simulated value [Fig. 11(b)]. The MV‐imager vertical parameter was also adjusted to 40 and 45 cm to acquire additional test points. The CPS predicted collision locations were all found to occur at a position before the actual collision was observed in room. The average prediction error for MV‐imager collisions was 1.5 cm.

#### 3.A.4 kV‐imager and kV‐source collisions

**Fig. 12:**
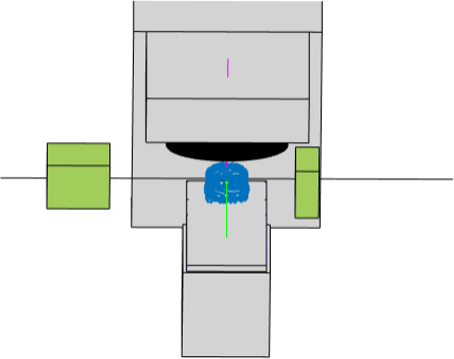
CPS Visualization of kV Imager and kV source models.

The kV‐imager and kV‐source models were tested using the same approach as the MV imager. The gantry was kept static at several different angles with the kV‐imager panel extended out at kV Vertical positions ranging from 40 to 55 cm. The couch was moved laterally until there was collision. A similar set of experiments were conducted for kV source. Since the number of test cases for the kV imager and source was not as exhaustive, the results have not been displayed graphically. Figure 12 displays the CPS visualization of the kV‐imager and kV‐source models. The average error in prediction was 4.2 and 1.3 cm for the kV imager and kV source, respectively.

### 3.B Evaluation of Kinect camera patient model

The Kinect camera scan data were scaled and transformed using a combination of the calibration procedure, described in section 2.B, as well as the ICP algorithm to match the Kinect scan with the planning CT body contour. This methodology minimizes the differences between the Kinect point cloud and the planning CT point cloud of the body. Since the planning CT is already an established baseline that is clinically acceptable, a reasonably strong registration between the two‐point clouds should be sufficient for the CPS’s purposes. Due to inherent limitations and error in the Kinect camera’s depth perception capabilities, the Kinect point cloud has some inaccuracies which have been documented in the past.^30^ These inaccuracies result in an imperfect registration with the planning CT body contour, however, the discrepancy between the two‐point clouds was not significant enough to effect the CPS performance.

### 3.C Evaluation of Couch coordinate prediction system

**Fig. 13:**
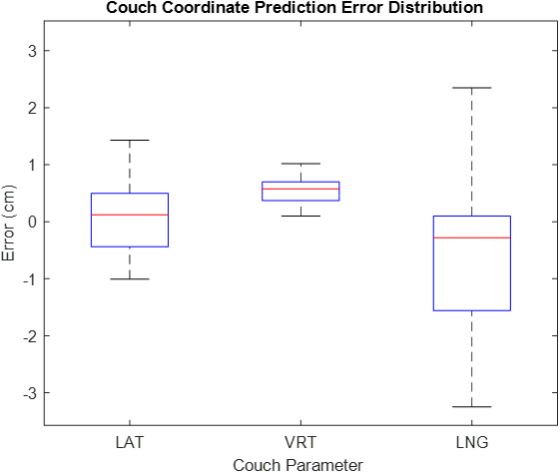
Box and whisker plot illustrating the distribution of error in Linac couch coordinate prediction for 18 patients across various treatment sites.

The performance of the couch coordinate prediction approach has been tested by selecting treatment plans for a wide variety of patients, and subsequently predicting the Linac couch coordinates. The predicted couch coordinates were then compared with the actual couch coordinates that were acquired during the first fraction of treatment. Figure 13 displays the prediction errors across the 18 patients tested. The average error in couch LAT, VRT, and LNG was 0.6, 0.7 and 1.1 cm, respectively.

### 3.D CPS end‐to‐end testing results

**Fig. 14:**
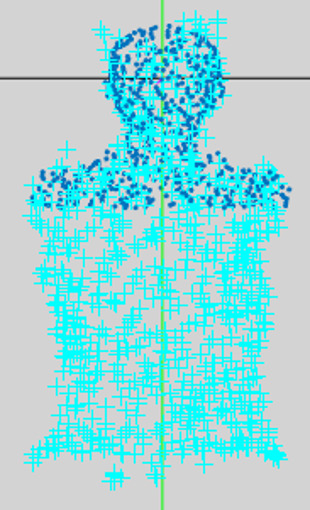
CPS Visualization of Patient model of Rando phantom for End to End testing (Case 1). Light blue points are kinect point cloud and dark blue poits are CT body contour point cloud.

To evaluate the CPS as a whole, end‐to‐end testing was done using a Rando phantom. The CPS workflow which includes acquiring a planning CT, Kinect Camera scan, selection of treatment isocenter, and prediction of couch coordinates has been tested. The Rando phantom was set up in the CT simulation room just like a regular patient. The phantom was set up on the CT couch so that the lasers specified a location at the center of the head of the phantom. BBs were placed on the phantom to specify the placement of the lasers and subsequently the user origin. Prior to acquiring the CT scan, a Kinect camera image of the Rando phantom was acquired. A typical head and neck protocol was followed while acquiring the CT scan of Rando. Once the CT was imported into the treatment planning system (Eclipse), the user origin was automatically identified. At this stage, the following test cases were designed:

Case 1: Treatment isocenter same as user Origin (as specified by BBs). Figure 14 illustrates the patient point cloud.

Case 2: Treatment isocenter shifted laterally with respect to user origin – Treatment isocenter (5, 0, 0).

Case 3: Treatment isocenter shifted longitudinally with respect to user origin – Treatment isocenter (0, 0, −5).

Case 4: Treatment isocenter shifted vertically with respect to user origin – Treatment isocenter (0, 5, 0).

Case 5: Treatment isocenter shifted in all directions with respect to user origin – Treatment isocenter (−3, −6, 1).

**Fig. 15:**
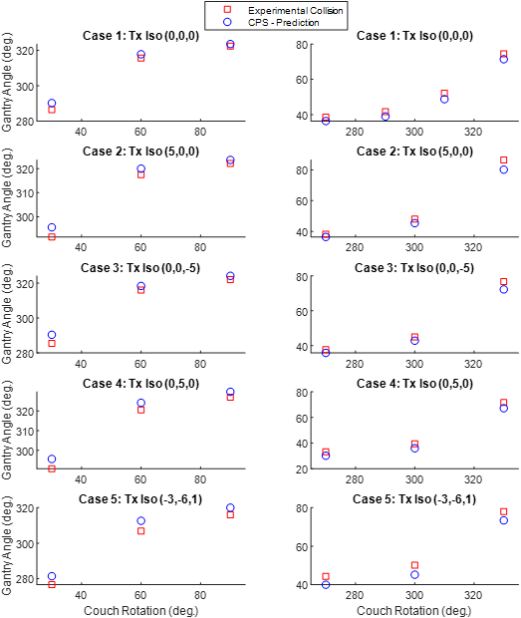
End to end testing of CPS for 5 different test cases. For each test case, the couch was rotated to specific angle and the gantry was rotated to a near collision state with Rando phantom. If needed, see Fig. 8 for reference diagram of common Linac motion parameter conventions.

For each test case, a separate DICOM plan file and structure file were exported so that the body contour for the part of the phantom that CT scanned can be imported into the CPS model. The selected treatment isocenter was used to calculate the predicted couch coordinates using Roswell Park Cancer Center’s in‐house couch prediction tool. The Rando phantom was then setup in the treatment room on the Linac couch use the same set up used in the CT simulation room. The couch position was adjusted to the predicted couch coordinates for the particular case being tested. At this point, the gantry was then rotated to preselected angles of 30, 60 90, 330, 300, and 270. For each static gantry angle, the couch was rotated until there was a collision. The couch rotation value was recorded and compared with the CPS predicted couch rotation value for the same set of parameters. The results of these end‐to‐end tests all had predictions in the conservative direction; no false negatives were observed (See Fig. 15). The average CPS prediction error across all five test cases was 3.4° with a standard deviation of 1.3°. The average error and standard deviation for each test case have been summarized in Table 2.

**Table 2:** Prediction error for CPS end‐to‐end testing for different choices of treatment Isocenter.

Test Case	Average prediction error (deg.)	Standard Deviation Of Error (deg.)
Case 1: Tx Isocenter – (0, 0, 0)	2.6	0.9
Case 2: Tx Isocenter – (5, 0, 0)	3.1	1.8
Case 3: Tx Isocenter – (0, 0,‐5)	3.0	1.3
Case 4: Tx Isocenter – (0, 5,0)	3.7	0.8
Case 5: Tx Isocenter – (−3,‐6,1)	4.8	0.6

### 3.E CPS performance metric

The overall predictive performance of the CPS was evaluated using the ROC formalism as specified earlier. The CPS prototype was tested over 111 test cases. The positive predictive value was calculated to be 0.95, while the negative predictive value was calculated to be 1. Table 3 summarizes the performance metric.

**Table 3:** CPS prototype performance evaluation.

	Number of test cases
True positive	88
False positive	5
True negative	18
False negative	0

## 4 DISCUSSION

The primary aim of the CPS framework has been to provide the treatment planner (dosimetrist) the ranges of gantry angles, corresponding to allowable ranges of couch positions (translation and rotation) to design a treatment plan that considers maximum possible noncoplanar beams. To accomplish this, the CPS can be incorporated into a normal clinical workflow. The systematic data required for the prototype CPS have been derived from geometric information of the relevant Linac components, in addition to a correspondence map between the CT couch and Linac couch. The patient‐specific data were derived from the CT scan data, and concurrently captured whole‐body Kinect camera images during the simulation session. Using these data, and by incorporating the location of the plan isocenter, as chosen by the dosimetrist, the CPS can provide the collision‐free zones (combination of couch positions and gantry directions).

In contrast to previous studies on collision detection, the CPS uses an easy to understand collision detection algorithm using the principle of dot products between vectors, and basic geometry. Therefore, the CPS framework can be easily tailored to meet specific needs in a wide range of clinical situations. The approach used in the CPS can be used to model SRS cones, electron cones, and different types of patient accessories. Furthermore, the methods can also be applied to other radiation therapy modalities where collisions are of concern, such as in cyber knife and proton therapy.

Some of the more recent works on collision detection such as Yu et al. have developed highly accurate systems using detailed CAD design models provided by the manufacturer and used hand‐held 3D scanners to acquire detailed patient models.^14^ Although this work has demonstrated the feasibility of predicting deliverable beams of individual patients for noncoplanar delivery, all aspects of the full clinical implementation of such a system have not been clearly discussed. The CPS framework developed in this investigation addresses three major challenges to clinical implementation that previous literature did not explore: (a) Couch coordinate prediction in the context of the clinical workflow, (b) A method to account for interfractional patient positioning variations using a built in margin, and (c) Options to verify patient positioning using imaging after couch rotations are applied.

In current clinical practice, KV or MV imaging is used to verify patient’s position prior to couch rotation for noncoplanar treatments. This approach relies on the assumption that after the couch rotation has been applied, the patient will be in the same position on the couch. For more accurate delivery, patient position should be verified after the couch rotation has been applied via imaging. Using a tool similar to the CPS described in this work, one can determine which angles are available for kV or MV imaging and subsequently plan on verifying patient positioning prior to radiation delivery at different available couch angles.

The collision prediction approach employed by Cardan involved a high‐degree complexity with the use of three Kinect Cameras as well as a fast polygon interference algorithm.^15^ In contrast, the prototype CPS framework uses only a single Kinect Camera. Although it is possible to obtain more detailed patient morphology with three cameras, there is an additional degree of complexity with registering 3D data from separate cameras. Furthermore, studies investigating the use of multiple Kinect cameras have demonstrated that there can be interference between the infrared signals from different cameras resulting in distortions.^31^ Our work demonstrates that using a single Kinect camera can be sufficient for collision prediction purposes and therefore the complexity of using multiple cameras can be avoided.

### 4.A CPS performance results

The CPS has three major components: (a) Geometric Model of the Linac, (b) Patient Model, and (c) Couch coordinate prediction. Each component of the CPS has been evaluated independently. Furthermore, the end‐to‐end testing of the entire CPS workflow using Rando demonstrates the feasibility of the overall framework.

While it is recognized that the separate components of the CPS contain their own uncertainties and subsequently contribute to the overall uncertainty of the CPS performance, it was deemed outside the scope of this feasibility study to analyze and quantify them explicitly. Prior to clinical implementation of a CPS, it is highly recommended that a thorough systematic uncertainty analysis be performed.

### 4.B Potential for CPS to improve workflow efficiency

In current clinical practice, dosimetrists rely on their clinical experience to select couch angle and gantry angle combinations when developing a treatment plan. With this approach there are two possible implications: (a) The dosimetrist does not consider all allowable collision‐free configurations of gantry and couch angles (specific to the patient being treated), thereby resulting in a suboptimal plan and (b) The dosimetrist may also unintentionally develop a nondeliverable plan due to potential collisions, thereby resulting in replanning. Both of these implications highlight an inefficiency in the current standard clinical workflow. The goal of developing a comprehensive CPS was to provide a solution that can help overcome these inefficiencies.

**Fig. 16:**
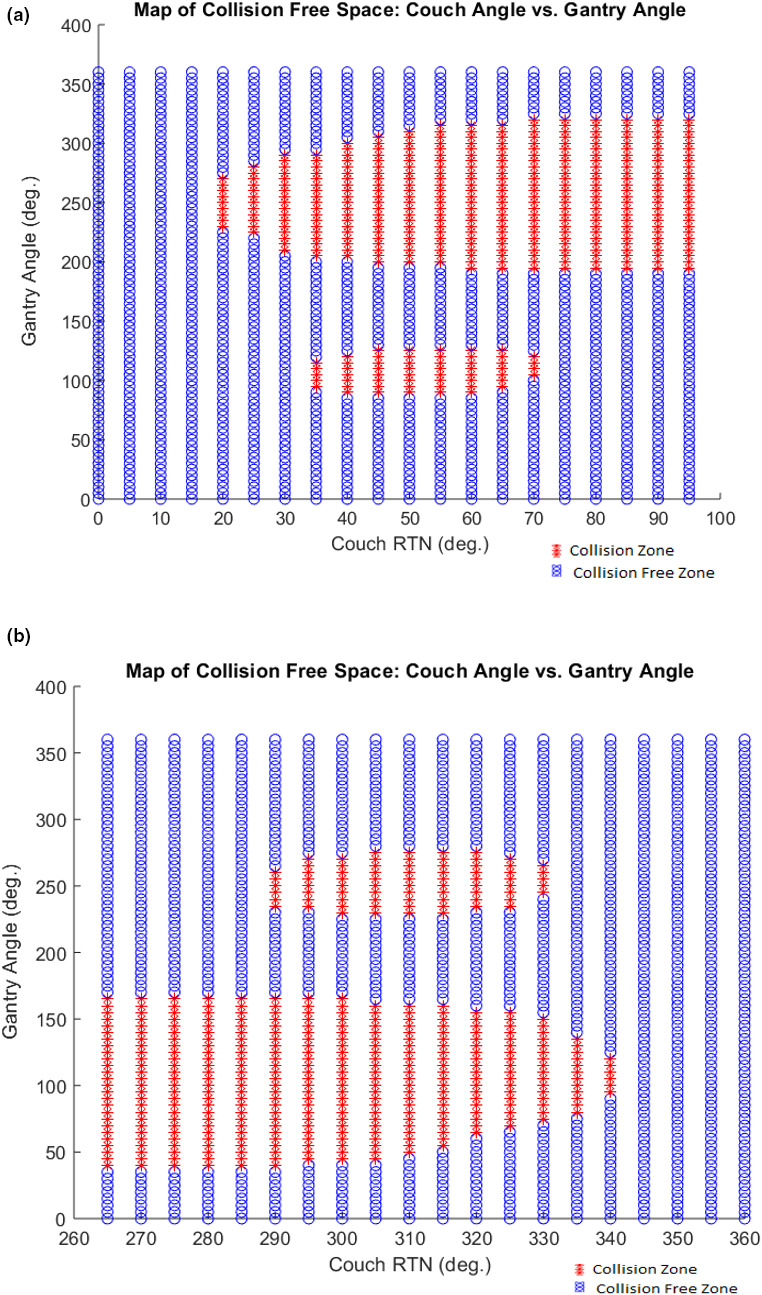
(a) Example CPS Output: Map of Collision Free Space for Rando Phantom. Treatment Isocenter = (0,0,0), Predicted Couch Coordinates: LAT = 999.16, LNG = 98.04, VRT = 10.42. If needed, see Fig. 8 for reference diagram of common Linac motion parameter conventions. (b) Example CPS Output: Map of Collision Free Space for Rando Phantom. Tx Isocenter = (0,0,0), Predicted Couch Coordinates: LAT = 999.16, LNG = 98.04, VRT = 10.42. If needed, see Fig. 8 for reference diagram of common Linac motion parameter conventions.

The overall performance results of the CPS demonstrate that the tool developed has an acceptable level of accuracy so that it can be incorporated into routine clinical workflow. For a given patient, once the planning CT has been acquired and the treatment isocenter has been selected, the CPS can then output an extensive graphical display of combinations of couch rotation and gantry rotation positions that are collision free. On a PC equipped with an Intel Core i5‐7200U CPU, 16 GB DDR3 RAM, running Windows 10 OS, it took approximately 88 seconds each to generate the data presented in Figs. 16(a) and 16(b), which display examples of such an output for the Rando phantom.

This visual display can serve as guidance, when the dosimetrist is designing a regular treatment plan with coplanar beams as well as noncoplanar beams. To fully take advantage of the CPS output from a treatment planning perspective, these data can be automatically input into treatment planning system so that it can be used during the plan optimization process.

In addition to providing the treatment planner with some confidence while generating plans with noncoplanar optimizations, the CPS can also provide a postplan generation virtual simulation to examine collision potential. The CPS is capable of iterating through control points and verifying that no collision is expected. While the CPS provides guidance for noncoplanar planning and additional checks for plans that have already generated, it is not intended to eliminate standard clinical safety protocols where a dry run is conducted with the patient just before treatment delivery. The use of CPS will reduce the occurrence of plan rejection and need for replanning due to collisions.

For clinics that may have already implemented noncoplanar planning and delivery techniques, the CPS can serve as a valuable secondary check that is independent from the treatment planning system.

### 4.C Considerations for clinical implementation of CPS

#### 4.C.1 Kinect camera

For routine clinical use, the Kinect Camera should be permanently mounted in the CT simulation room, preferably on the ceiling with a direct view of the entire CT couch. In that scenario, the calibration procedure involving the plastic block used in our prototype study may not be suitable due to the orientation of the camera. Therefore, a different phantom will be needed to be designed to help transform the Kinect camera data into the CPS coordinate system accurately.

To improve the accuracy of the Kinect Patient model, the depth sensing capability of the Kinect Camera should also be calibrated using an independent procedure. There are various documented methods available, such as using a semi‐transparent checkerboard pattern with different depths.^32^

#### 4.C.2 Patient positioning devices

In the present work, specialized patient immobilization devices were not tested in the CPS workflow. However, the framework developed in the CPS is fully capable of handling a wide range of patient support systems. The Kinect camera can be used to capture the support structures as part of the patient model. Furthermore, parts of the support structure captured in the planning CT can also be contoured and subsequently added to the CPS model.

#### 4.C.3 CPS geometric model accuracy

The CPS model by design contained extra margins with the goal of providing conservative collision prediction results. The cylinder used to model the gantry head was designed to encapsulate the minor structures (accessory tray structures: posts and pins) at the head of the collimator. Therefore, the base of the cylinder was modeled at the lowest structure on the collimator face that was closest to isocenter. Furthermore, the couch was modeled using a trapezoidal prism shape that also contained some conservative dimension assumptions to account for the tapering of the couch at various locations. A combination of these factors resulted in a conservative CPS model that contained a built‐in margin. Further refinement of the geometric models of the CPS can be applied to achieve a higher level of accuracy, however, such refinements should be done with consideration of the fact that a reasonable level of conservativeness is desirable for workflow efficiency.

In normal clinical practice, the patient position on the treatment couch can vary from day to day typically on the order of 1–2 cm. This variation in patient positioning is then reflected in a different set of treatment couch coordinates required to align the patient to treatment isocenter. This variation in day‐to‐day treatment couch coordinates is well documented in tolerance tables that are specific to treatment site. Most tolerances for couch position variation in all three directions were in the 1–3 cm range.^33^ Therefore, an inherent margin in the CPS model can help offset these typical variations in couch positions. The magnitude of the error in CPS prediction, which was consistently in the conservative direction, was similar to the typical tolerance in allowable couch position variance.

Aiming for a very accurate model will run the risk of collision potential during clinically acceptable day‐to‐day variations in patient positioning on the couch, and subsequent couch position changes. In contrast, aiming for a model with a conservative margin will result in a loss of allowable beam approach angles available for certain couch and gantry angle combinations. To summarize, prior to clinical implementation of the CPS, the clinician must decide on a reasonable balance between accuracy and maintaining a conservative margin to account for interfractional set‐up variations.

## 5 CONCLUSION

To summarize, this work has demonstrated the feasibility of incorporating an easy to understand collision prediction framework into the modern treatment planning workflow. The methods used to model the patient, linear accelerator, treatment couch, and imaging devices can be applied to a wide range of Linac models. The output from the CPS can serve as valuable reference for treatment planners and can result in not only more efficient workflow but can also result in the development of a dosimetrically superior plan. Furthermore, the CPS can also be used postplan generation to conduct secondary collision safety checks. The framework described in this study can serve as valuable reference to clinicians who seek to apply the same principles in developing their own in‐house collision prediction system.

## CONFLICTS OF INTEREST

The authors have no relevant conflicts of interest to disclose.
